# E-syt1 Re-arranges STIM1 Clusters to Stabilize Ring-shaped ER-PM Contact Sites and Accelerate Ca^2+^ Store Replenishment

**DOI:** 10.1038/s41598-019-40331-0

**Published:** 2019-03-08

**Authors:** Fei Kang, Mengxuan Zhou, Xiaoshuai Huang, Junchao Fan, Lisi Wei, Jerome Boulanger, Zengzhen Liu, Jean Salamero, Yanmei Liu, Liangyi Chen

**Affiliations:** 1 State Key Laboratory of Membrane Biology, Beijing Key Laboratory of Cardiometabolic Molecular Medicine, Institute of Molecular Medicine, Peking University, Beijing, 100871 China; 2 Key Laboratory of Image Processing and Intelligent Control of Ministry of Education of China, School of Automation, Huazhong University of Science and Technology, Wuhan, 430074 China; 3 Institut Curie, PSL Research University, CNRS UMR 144 & Cell and Tissue Imaging Facility, Paris, France

## Abstract

In many non-excitable cells, the depletion of endoplasmic reticulum (ER) Ca^2+^ stores leads to the dynamic formation of membrane contact sites (MCSs) between the ER and the plasma membrane (PM), which activates the store-operated Ca^2+^ entry (SOCE) to refill the ER store. Two different Ca^2+^-sensitive proteins, STIM1 and extended synaptotagmin-1 (E-syt1), are activated during this process. Due to the lack of live cell super-resolution imaging, how MCSs are dynamically regulated by STIM1 and E-syt1 coordinately during ER Ca^2+^ store depletion and replenishment remain unknown. With home-built super-resolution microscopes that provide superior axial and lateral resolution in live cells, we revealed that extracellular Ca^2+^ influx via SOCE activated E-syt1s to move towards the PM by ~12 nm. Unexpectedly, activated E-syt1s did not constitute the MCSs *per se*, but re-arranged neighboring ER structures into ring-shaped MCSs (230~280 nm in diameter) enclosing E-syt1 puncta, which helped to stabilize MCSs and accelerate local ER Ca^2+^ replenishment. Overall, we have demonstrated different roles of STIM1 and E-syt1 in MCS formation regulation, SOCE activation and ER Ca^2+^ store replenishment.

## Introduction

Various cellular organelles communicate via MCSs that mediate important cellular processes, such as intracellular signaling, metabolism, metabolite trafficking and organelle division^1^. Upon depletion of Ca^2+^ stores, STIM1 on the ER membrane changes configuration to form transient MCSs via its interaction with Orai1 on the PM^2^, which opens Ca^2+^ release-activated Ca^2+^ (CRAC) channels to mediate the store-operated Ca^2+^ entry (SOCE)^3^. Subsequently, Ca^2+^ influx via SOCE activates E-syt1 to dock ER onto the PM that’s enriched with phosphatidylinositol 4,5-bisphosphate (PIP_2_), culminating in the formation of new ER-PM contacts^4–6^. E-syt1-MCSs appear partially co-localized with STIM1 in live HeLa cells upon ER Ca^2+^ store depletion and replenishment^5^, and catalyze phospholipid shuttling between ER and PM that mediates the sustained activation of the receptor-induced Ca^2+^ signaling pathway^6^ and the slow Ca^2+^-dependent inactivation of the CRAC current^7^. However, arguing against a collaborative role of E-syt1 and STIM1 in inducing MCSs, knockdown of E-syt1 failed to reduce SOCE^4^, distinct from the essential role of STIM1 in the activation of SOCE^8,9^. Under an electron microscope, the ER membrane in the MCSs formed by E-syt1 is ~15 nm away from PM, while the ER membrane in the MCSs formed by STIM1 are further away from the PM (~22 nm)^6,10^, despite no dynamic information is available. Conventional live fluorescence imaging techniques, on the other hand, do not have sufficient spatiotemporal resolution to resolve MCSs that have an average size of ~100 nm in the lateral direction^11^. Although super-resolution imaging techniques were used to explore the structure of MCSs^12^, the prerequisite need for excessive photons has prevented the unequivocal visualization of the *in vivo* assembly and disassembly of ER-PM MCSs in live cells.

## Results

**Figure 1:**
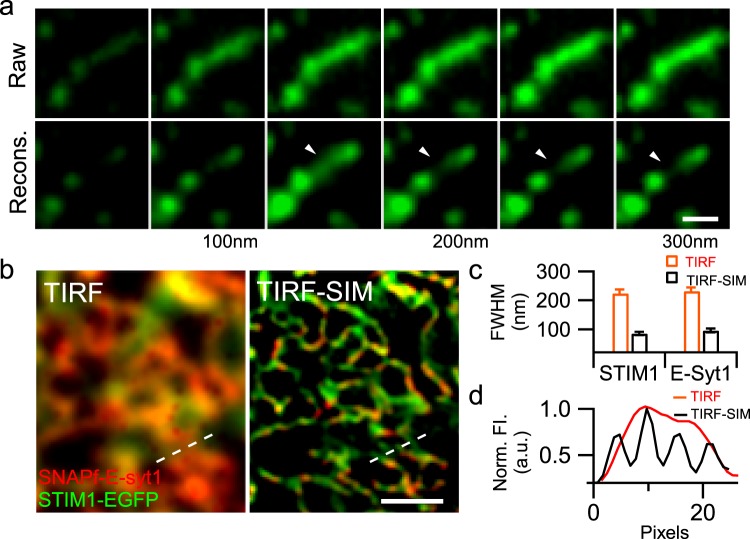
Live-cell imaging of cortical ER in HEK293 cells using VA-TIRFM or TIRF-SIM. (**a**) The HEK293 cell was transfected with mCherry-Sec 61β and observed using VA-TIRFM. Montages show TIRF planes before (Raw) and after reconstruction (Recons.), which differ by an increasing penetration depth of 50 nm from left to right. We identified fluorescent puncta only in the 150 nm plane (arrowhead) but not in the planes above or below, indicating an axial resolvability of 50 nm. (**b**) The same image under the TIRF (left) and TIRF-SIM (right). The HEK293 cell transfected with SNAPf-E-syt1 and STIM1-EGFP was observed with dual-color TIRF-SIM. (**c**) Resolution was calculated as the full-width half-maximum (FWHM) of the fluorescence profiles along the narrowest tubules (n = 43), as a representative example shown by the short white line in the right corner of (**b**). (**d**) Normalized fluorescence intensity along the dashed line at the bottom of (**b**), in which four closely packed tubules convolved into one large punctum under TIRF but were resolved individually under TIRF-SIM. Scale bars, (**a**) 0.5 μm; (**b**) 1 μm.

To address these problems, we have built a versatile variable-angle total internal reflection fluorescence microscopy (VA-TIRFM) system that enables live-cell super-axial-resolution volumetric imaging^13^, which allows to recover dynamic with a 3D volume from images obtained at continuously varied incident angles, and another high-NA total internal reflection fluorescence structured illumination microscopy (TIRF-SIM) with the ~sub 90 nm lateral resolution at minimal photon budgets^14^. Using the VA-TIRFM system, we performed live-cell imaging of HEK293 cells expressing ER membrane-located red fluorescent marker mCherry-Sec 61β at different incident angles under the TIRF mode^13,15^. The raw images were then reconstructed using a previously developed algorithm^16^ and rendered in Amira (Thermo Scientific, USA) to enable three-dimensional structure visualization (Detailed in the Material and Methods, and in Supplementary Fig. 1, Video S1). Using this method, we could identify mCherry-Sec 61β labeled ER structure in the layer 100–150 nm beneath the PM (Fig. 1a) but not in the layers one section above (50–100 nm) or below (150–200 nm), indicating an axial resolvability of 50 nm. On the other hand, with the high-NA, dual-color TIRF-SIM, we could achieve a lateral resolution of ~85 nm and 100 nm for EGFP and 647-SiR (to label SNAPf-E-syt1, Fig. 1b,c), which helps to resolve a large fluorescent puncta in the TIRF to be four parallel-arranged ER tubules (Fig. 1d).

**Figure 2:**
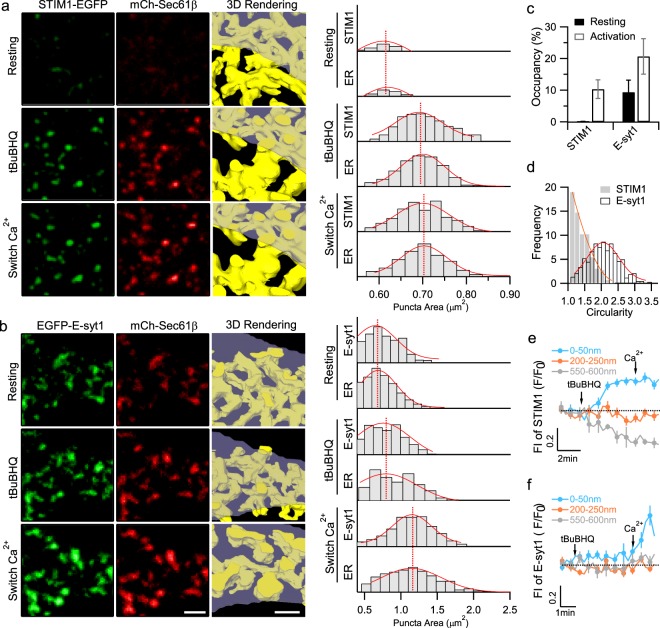
Distinct morphology and kinetic assembly of the ER-PM MCSs mediated by STIM1 or E-syt1 under VA-TIRFM. (**a**,**b**) Representative planes at 0–50 nm and volumetric reconstruction of cortical ER in HEK293 cells co-transfected with mCherry-Sec 61β and STIM1-EGFP (**a**) or EGFP-E-syt1 (**b**) under resting conditions (upper left), upon treatment with tBuBHQ (middle left, store depletion) and after changing to a Ca^2+^-containing HBSS bath solution (lower left, store replenishment). Histograms of the sizes of the STIM1, E-syt1, and Sec 61 puncta under resting conditions, upon treatment with tBuBHQ and after switching the bath solution to a Ca^2+^-containing HBSS are shown in the right panels. Data are representative of three independent experiments. Scale bars, 2 μm. (**c**) The percentage of the cell surface occupied by the STIM1-EGFP or EGFP-E-syt1 puncta under resting conditions, upon treatment with tBuBHQ and after switching the bath solution. (**d**) Circularity analysis of mCherry-Sec 61-indicated ER-PM contacts in the cells co-transfected with STIM1-EGFP or EGFP-E-syt1 after SOCE activation. Circularity with a value of 1.0 indicates a perfect circle. (**e**,**f**) Time courses of normalized fluorescence signals of STIM1 (**e**) and E-syt1 (**f**) at the planes 0–50 nm, 200–250 nm or 550–600 nm beneath the PM under VA-TIRFM. Mean ± SEM is shown (n = 8 from three independent experiments).

Having established these methods, we monitored the changes in the ER structures within a 0–50 nm depth beneath the PM using our VA-TIRFM. In resting HEK293 cells co-expressing STIM1-EGFP and mCherry-Sec 61β, we observed almost no ER structures in the layer 0–50 nm beneath the PM (Fig. 2a,b). We used 2,5-di-(t-butyl)-1,4-benzohydroquinone (tBuBHQ), which is a reversible sarcoplasmic/ER Ca^2+^-ATPase (SERCA) inhibitor, to deplete the ER Ca^2+^ store in a Ca^2+^-free bath solution, followed by store replenishment via SOCE by switching to a bath solution containing 1.26 mM CaCl_2_^5^. Upon ER Ca^2+^ store depletion, STIM1 gradually aggregated to induce new ER-PM MCSs formation, as indicated by the increased number and size of the STIM1-EGFP and mCherry-Sec 61β, which remarkably co-clustered within 50 nm beneath the PM in the raw images, and the images rendered by volumetric imaging (Fig. 2a) and quantification analysis (Fig. 2c). However, subsequent Ca^2+^ entry via SOCE failed to induce a further increase in either the number or the size of MCSs (Fig. 2a).

E-syt1-mediated ER-PM MCSs were already partially appeared under resting conditions and the depletion of the ER Ca^2+^ store led to a subtle accumulation (Fig. 2b). Nevertheless, subsequent activated Ca^2+^ influx via SOCE triggered a massive expansion in both the number and size of MCSs (Fig. 2b), which occupied up to 20% of the cell membrane after the E-syt1 activation (Fig. 2c). The shapes of the E-syt1-mediated MCSs were more irregular than those of the STIM1-mediated MCSs (Fig. 2d). To quantify the stimulation-evoked axial movements of the E-syt1 and STIM1 proteins, we simultaneously plotted fluorescence signals from different layers throughout the entire process. Upon the ER Ca^2+^ store depletion, the increases in the STIM1 fluorescence in the layer 0–50 nm beneath the PM were parallel with the decreases in the STIM1 fluorescence in the deepest layer observed (550–600 nm beneath the PM) with similar kinetics, while the STIM1 fluorescence in the middle layer (200–250 nm beneath the PM) remained nearly unchanged (Fig. 2e). In contrast, following Ca^2+^ influx, the EGFP-E-syt1 signals in the deeper layers did not change, while the signal intensity increased in the layer 0–50 nm beneath the PM (Fig. 2f). Altogether, our results suggest that STIM1 migrates from the internal ER tubules to the cell surface to mediate STIM1-MCS formation upon ER Ca^2+^ store depletion, while Ca^2+^ influx induces a further approach of E-syt1 molecules that were previously docked at the cell periphery towards the PM to form E-syt1-MCSs. In activated E-syt1 puncta exhibiting increases in fluorescence intensity with no apparent protein aggregation, SOCE triggered an increase of fluorescence intensity by 27 ± 6% (n = 23). Assuming an exponential decay of TIRFM excitation determined by the illumination angle, such changes of fluorescence intensity corresponded to an axial movement of 12 ± 2 nm (explained in detail in Material and Method), consistent with previous reports^4,6,10^. Therefore, MCSs mediated by STIM1 or E-syt1 differ in formation kinetics, morphology and mechanism.

**Figure 3:**
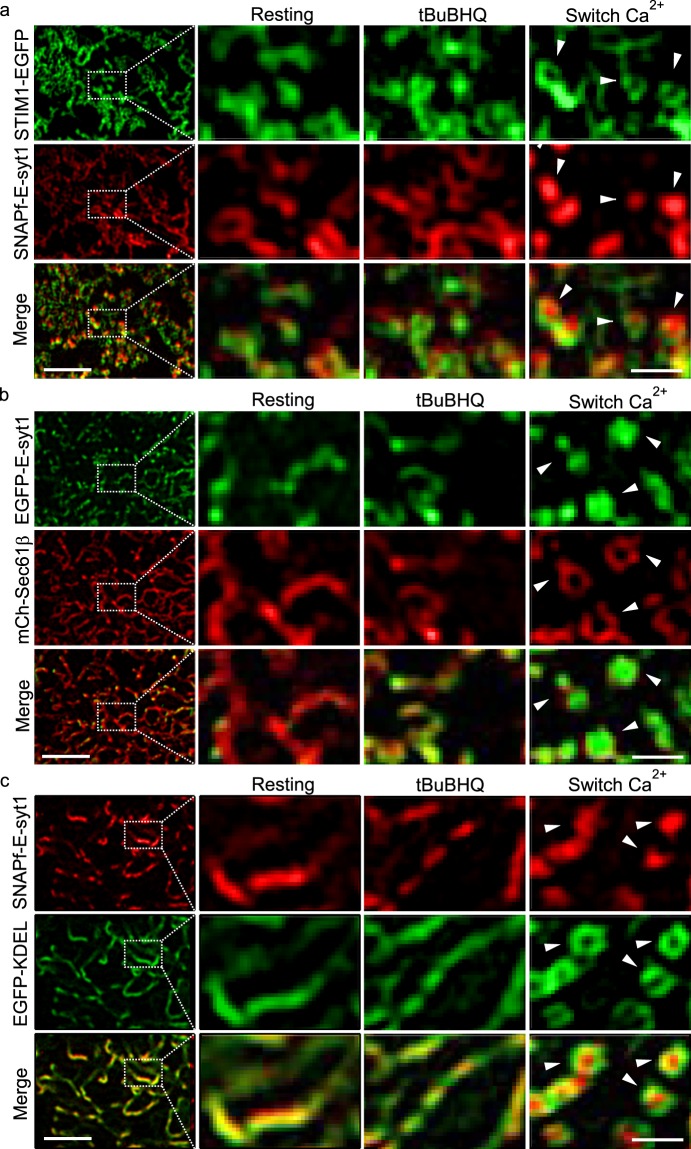
Ca^2+^ influx via SOCE activates E-syt1 to reshape punctate ER-PM MCSs into ring-shaped structures under TIRF-SIM. (**a**–**c**) Representative TIRF-SIM images of cortical ER in HEK293 cells co-transfected with SNAPf-E-syt1 and STIM1-EGFP (**a**), EGFP-E-syt1 and mCherry-Sec 61β (**b**), or SNAPf-E-syt1 and EGFP-KDEL (**c**). The left panels show images of cells under resting conditions at a lower magnification; boxed areas are magnified and shown on the right. From left to right are cells under resting conditions, store depletion and store replenishment. All these figures are representative of four independent experiments. Scale bars, left 2 μm; right 0.5 μm. Related videos were also shown as Supplementary Videos S2–S4.

Next, we examined how STIM1 and E-syt1 interact with each other during Ca^2+^ store depletion and replenishment in HEK293 cells co-transfected with both STIM1-EGFP and SNAPf-E-syt1. Under regular TIRFM, STIM1 and E-syt1 mostly co-localized regardless of whether the cells were in resting state, or after store depletion or subsequent SOCE activation (Supplementary Fig. S2). However, as VA-TIRFM did not provide sufficient resolution in the lateral axis, it may not accurately differentiate STIM1 and E-syt1 puncta under different experimental conditions. TIRF-SIM, on the other hand, allows to observe that STIM1 but not E-syt1 puncta form upon Ca^2+^ store depletion (Fig. 3a). Within the first tens of seconds after the initiation of SOCE, these STIM1 puncta, before complete disassembly, quickly rearranged into ring structures enclosing the emerging E-syt1 puncta, which have never been observed before (arrowheads, Fig. 3a, Supplementary Video S2). If HEK293 cells were transfected with STIM1 and Sec 61β, then STIM1 puncta evoked by store depletion remained unchanged after switching to a Ca^2+^-containing solution (Supplementary Fig. S3)^8,9,17^, indicating that STIM1 alone does not mediate ring-shaped MCS formation. In contrast, in HEK293 cells overexpressing EGFP-E-syt1 and mCherry-Sec 61β, the Ca^2+^ influx-induced EGFP-E-syt1 puncta were also surrounded by mCherry-Sec 61β rings that emerged during SOCE activation (Fig. 3b, Supplementary Video S3). Moreover, EGFP-KDEL, a canonical probe to label the ER lumen, also exhibited ring-shaped structures enclosing the E-syt1 puncta upon Ca^2+^ store replenishment (Fig. 3c). Collectively, these data suggest that the E-syt1 help to re-arrange neighboring ER structures into ring-shaped MCS, but do not constitute the ER-PM contacts *per se*. Interestingly, the STIM1 rings (230 ± 22 nm in diameter, n = 112) were smaller than the Sec 61β and KDEL rings (Sec 61β: 282 ± 31 nm, n = 131, p < 0.05; KDEL: 279 ± 28 nm, n = 94, p < 0.05, Fig. 3C and Supplementary Fig. S4, Video S4), indicating that STIM1 proteins were preferentially located at the innermost edges of the rings, and may function collaboratively with E-syt1 to form the ring-shaped MCSs.

**Figure 4:**
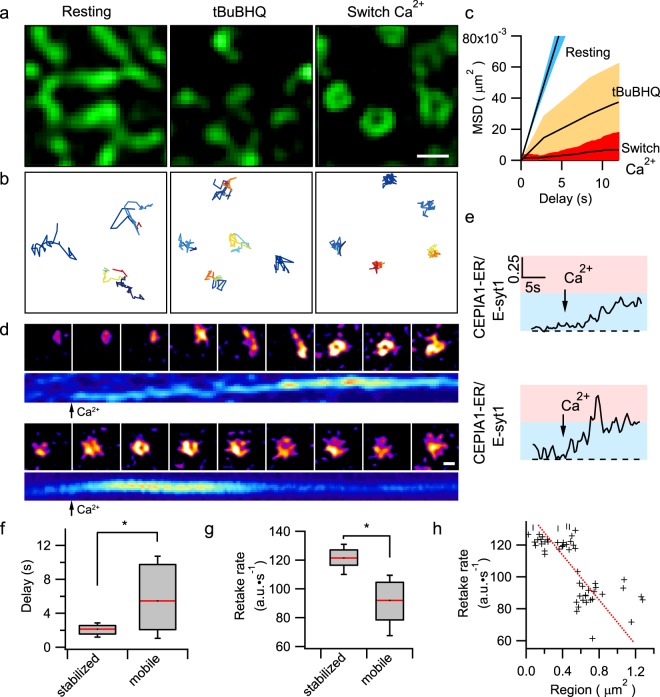
E-syt1 stabilizes ER-PM contacts to facilitate rapid ER Ca^2+^ store replenishment. (**a**,**b**) Representative TIRF-SIM images (**a**) and tracking trajectories of MCSs (**b**) of cortical ER in HEK293 cells co-transfected with SNAPf-E-syt1 and STIM1-EGFP under resting conditions, store depletion and store replenishment (as an example shown in Supplementary Video S5). (**c**) MSD analysis of cortical ER indicated by STIM1-EGFP under different conditions as shown in a. (**d**) Montages and kymographs of two representative examples of G-CEPIA1er labeled cortical ER during the store replenishment process. Top: A representative punctum initially traveled and ultimately became stationary after the application of Ca^2+^-containing solution, Bottom: A representative punctum kept stationary from the beginning. Kymographs show the dynamic local ER Ca^2+^ uptake at corresponding sites. HEK293 cells were co-transfected with EGFP-E-syt1 and G-CEPIA1er. (**e**) Time courses of the normalized G-CEPIA1er signals (in reference to SNAPf-E-syt1) in the two events in d. (**f**) Time delays between the application of Ca^2+^ and the increase in G-CEPIA1er fluorescence at the initially stabilized or mobile MCSs. (**g**) Average maximum rate of local ER Ca^2+^ uptake at initially stabilized (n = 32) or mobile (n = 35) MCSs. (Three independent experiments, and the asterisks denote significant differences, p < 0.05) (**h**) Correlation between the maximum rate of local ER Ca^2+^ uptake at MCSs and the area around which MCSs had traveled. The red dashed line indicates an inverse linear aggression of the ER uptake rates to area of MCS movements. Scale bars, (**a**) 0.5 μm; (**d**) 0.25 μm.

To examine the consequence of MCS re-arrangements from punctate to ring-shaped structures, we tracked STIM1-EGFP labeled ER structures close to the PM and analyzed their trajectories under different conditions in cells co-transfected with SNAPf-E-syt1. In resting cells, the cortical ER moved randomly and rigorously, as shown by the large time-dependent linear increase in the mean squared displacement (MSD) (Fig. 4A,B). Using tBuBHQ to deplete the ER Ca^2+^ store, STIM1 formed punctate MCSs, which resulted in overall reductions in the ER mobility with a smaller MSD (<0.92 μm^2^) (Fig. 4c). The extracellular Ca^2+^ entry further transformed punctate ER-PM MCSs into ring shaped structures which became even more restricted in motion (MSD < 0.41 μm^2^) (Fig. 4c, also Supplementary Video S5). Therefore, following sequential ER store depletion and replenishment, MCSs become more stably anchored to designated sites.

Finally, we explored whether these stable MCSs facilitate cellular and ER Ca^2+^ regulation. We either loaded HEK293 cells with the fluorescent calcium indicator Cal-520 or transfected cells with the gene-encoded ER Ca^2+^ indicator G-CEPIA1er^18^. When compared to control cells, overexpression of STIM1 alone potentiates maximal Ca^2+^ transients evoked by SOCE, but did not affect tBuBHQ-stimulated ER Ca^2+^ store release or rate of ER Ca^2+^ uptake (Supplementary Fig. S5A). On the other hand, consistent with the lack of an effect of E-syt1 on cytosolic Ca^2+^ transients^4,5^. tBuBHQ-stimulated ER Ca^2+^ store release and Ca^2+^ influx via SOCE were indistinguishable between control cells and cells transfected with E-syt1, or between cells overexpressing STIM1 and those overexpressing both STIM1 and E-syt1 (Supplementary Fig. S5A, B). Paradoxically, during store replenishment the ER Ca^2+^ concentration in cells expressing E-syt1 exhibited a much faster rise than cells without E-syt1 overexpression, including those cells with up-regulated SOCE due to STIM1 overexpression (Supplementary Fig. S5C, D). Given that the transfection with E-syt1 did not increase the amplitude or the speed of SOCE *per se*, we wondered whether the accelerated replenishment of the ER Ca^2+^ store was due to the more stably tethered ER-PM MCSs. Therefore, we used TIRF-SIM to examine the local ER Ca^2+^ dynamics at the MCSs in cells co-transfected with SNAPf-E-syt1 and G-CEPIA1er (Fig. 4d,e, Supplementary Fig. S6), and normalized the fluorescence intensities of G-CEPIA1er by those of SNAPf-E-syt1 at the same location to remove the effect of axial movements on the G-CEPIA1er signal (Supplementary Fig. S6B). As shown in the representative examples in Fig. 4d,e, the Ca^2+^ levels in the mobile ER tubules and puncta were relatively unchanged even in the presence of Ca^2+^ influx via SOCE. However, once the mobile E-syt1 puncta became stationary and formed ring structures, the luminal Ca^2+^ levels in the corresponding spots increased and manifested as either abrupt Ca^2+^ transients or slow increases in Ca^2+^ levels (Fig. 4e). On average, the delay between the addition of Ca^2+^ to the bath solution and initiation of the increase in the ER Ca^2+^ level in initially stationary G-CEPIA1er structures was significantly less than that in the initially mobile structures (Fig. 4f). Moreover, the ER Ca^2+^ uptake rates in the stationary G-CEPIA1er structures were higher than those in the mobile structures (Fig. 4g), and these rates were inversely correlated with the size of the region enclosing the MCS movement (Fig. 4h). Altogether, stably anchored MCSs surrounding E-syt1 puncta replenish the ER Ca^2+^ store more rapidly and locally with the same amount of Ca^2+^ influx rate via SOCE.

## Discussion

**Figure 5:**
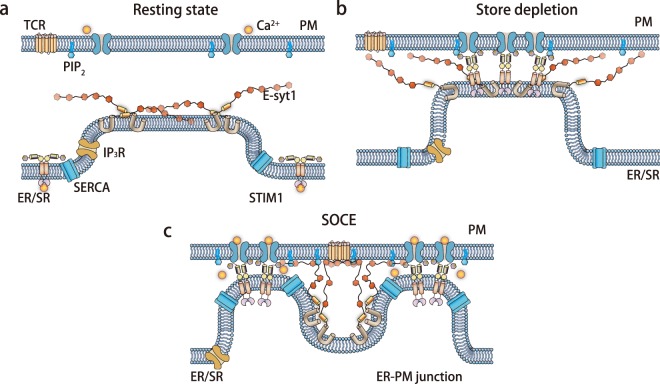
A model of how E-syt1 and STIM1 collaboratively and dynamically regulate ER-PM MCSs. (**a**) Resting state; (**b**) store depletion; (**c**) store replenishment.

Based on our data and current knowledge, we propose the following model (Fig. 5): in non-excitable cells, the agonist- or antigen-mediated activation of G protein receptors on the PM increases phospholipid C activity, which hydrolyzes PIP_2_ to generate inositol trisphosphate to release ER Ca^2+^ stores. Upon partial ER Ca^2+^ store depletion, STIM1 begins to move toward the PM to form MCSs and activate SOCE on the PM. Subsequently, Ca^2+^ influx via SOCE triggers E-syt1 that reshapes the originally formed MCSs into ring structures, which was, for the first time, revealed by our live cell super-resolution microscopy system. MCS rings are closer to the PM and are more stable, which facilitates rapid replenishment of ER Ca^2+^ stores possibly via enhancing the Ca^2+^ translocation activity of SERCA proteins that are enriched also at the ER-PM MCSs^19,20^. Together with the Ca^2+^-dependent slow inactivation of CRAC channels attributed to the shuffling of PIP_2_ on the PM^7^, efficient ER Ca^2+^ store replenishment de-activates STIM1, closes the CRAC channels, and limits the duration of extracellular Ca^2+^ entry, thereby preventing possible Ca^2+^ overload and toxicity. Finally, de-activated STIM1 in rings may disassemble more rapidly than STIM1 puncta, as more STIM1 molecules are exposed to the surrounding environment in the ring structures (Fig. 5). Interestingly, it is known that knocking down endogenous E-syt1 delayed the clearance of cytosolic Ca^2+^ after the peak evoked by SOCE (Figure 7I in^4^). The mechanism was not known in the previous study^4^, but could be explained by the possible coupling between E-syt1 and SERCA, i.e. E-syt1 may contribute to ER Ca^2+^ store replenishment through regulating SERCA. Following the knockdown of E-syt1, pulsed histamine stimulation at 1 pulse/min triggered the damping of cytosolic Ca^2+^ transients over time (Figure 6G in^6^), which was hypothesized to be due to defective PIP_2_ replenishment^6^. As E-syt1 is important for the refilling of ER Ca^2+^ store (Figs 4 and S5), reduced endogenous E-syt1 may lead to incomplete ER refilling during sustained receptor activation and contribute to the gradually reduced ER Ca^2+^ release.

It is unexpected that the E-syt1 puncta evoked by Ca^2+^ influx were devoid of other ER components, including the membrane protein SEC 61β and luminal protein KDEL (Fig. 3). Because the cytosolic domain of E-syt1 is longer than that of STIM1^4,6^, interaction of E-syt1 with lipids on the PM may allow other short-length tethering proteins to collaboratively function in reshaping ER-PM MCSs. Another counter-intuitive finding is that the Ca^2+^ levels and dynamic changes among different ER-PM MCSs during store replenishment were relatively heterogeneous (Fig. 4d,e and Supplementary Fig. S6), which is hard to reconcile with the fast Ca^2+^ diffusion and equilibrium process within the ER lumen. The cortical ER can extend to the cell surface as narrow tubules of ~15 nm in diameter^10^. Moreover, the dense tubular matrices of the peripheral ER can be extremely dynamic^21^. Thus, narrow and fast-moving ER tubules connected to MCSs may constitute the diffusion barrier of luminal Ca^2+^ and create transient Ca^2+^ micro-domains at the ER-PM MCSs. Whether these compartmentalized ER structures play additional roles in restricting signaling pathways other than Ca^2+^ or creating insulated micro-domains enriched in lipids and protein complexes remains to be addressed in future.

In summary, here we have revealed a unique role of E-syt1 in structurally reshaping and stabilizing pre-existed STIM-mediated ER-PM MCSs, which enables rapid ER Ca^2+^ store replenishment by STIM1-dependent Ca^2+^ influx via SOCE. Although the present study is conducted in HEK cells with exogenously over-expressed E-syt1, the similar mechanism also operates in A2058 cells with endogenous E-syt1s during ER Ca^2+^ store replenishment (Fig. S7). Because E-syt1 is ubiquitously expressed^22^, the mechanism discovered here may also mediate ER-PM MCSs in other cell types, such as neurons^10^ and migrating cancer cells^23^. Besides STIM1 and E-syt1, there exist many other tethering proteins that mediate ER-PM MCSs^24^. One insight from our study is that the addition or loss of one tethering protein may profoundly change the local structure of MCSs to better coordinate reciprocal information flows, and these dynamic processes can be studied with live super-resolution imaging microscopy techniques.

## Material and Methods

### Cell culture and fluorescence labeling

HEK293 cells were originally purchased from ATCC, and have been used in several previous papers^25,26^. HEK293 cells of the passage number within 30 were used, and cultured in high-glucose Dulbecco’s Modified Eagle’s Medium (DMEM) without phenol red (Life Technologies) supplemented with 10% (vol/vol) fetal bovine serum (FBS), 100 U/ml penicillin and 100 U/ml streptomycin. A2058 (obtained from ATCC) cells were cultured in Dulbecco’s modified Eagle’s medium supplemented with 10% fetal bovine serum (Life Technologies, Inc.). The cells were grown at 37 °C in a humidified atmosphere of 5% CO_2_ and 95% air. The transient transfection was performed using Lipofectamine 2000 (Invitrogen) according to the manufacturer’s instructions. After the transfection, the cells were trypsinized and seeded onto poly-L-lysine-coated customized high refractive index coverslips.

The cells transfected with SNAPf-E-syt1 were labeled with 3 µM cell-permeable far-red SNAP-Cell 647-SiR (New England BioLabs, Catalog # S9102S, excitation/emission at 645/661 nm) at 37 °C and 5% CO_2_ for 30 min, washed three times with fresh medium and incubated for another 30 min before imaging.

### Coverslip preparation

To clean the custom-made coverslips (25 mm diameter, 0.15–0.17 mm thick) for the live-cell imaging, we immersed the coverslips in 10% Alconox (Sigma-Aldrich), followed by sonication for 20 min. After 8 rinses in deionized water, the coverslips were sonicated in acetone for 15 min and then sonicated again in 1 M KOH for 20 min. Finally, we extensively washed the coverslips with deionized water, followed by sonication for 10–15 min. The clean coverslips were stored in 95% ethanol at 4 °C.

### Reagents and antibodies

tBuBHQ was purchased from Alomone Labs. Primary antibodies: anti-STIM1 (ProteinTech) rabbit polyclonal antibody (11565-1-AP); anti-Esyt1 (Sigma-Aldrich) rabbit polyclonal antibody (HPA016858); mouse monoclonal anti-tubulin antibody (Sigma-Aldrich). Secondary Antibody: goat anti-rabbit IgG (H + L) cross-adsorbed secondary antibody, Alexa Fluor® 647 conjugate (A-21244); anti-rabbit-HRP (Santa Cruz).

### Plasmid constructs and subcloning

The plasmids EGFP-E-syt1^4^, mCherry-Sec 61β^27^ and G-CEPIA1er^18^ were obtained from De Camilli lab, Voeltz lab and Lino labs through Addgene. SNAPf-E-syt1 was generated by inserting the E-syt1 gene into a pSNAPf vector (New England BioLabs, Catalog # N9183S). All plasmid constructs used in this study were prepared according to standard molecular cloning approaches and validated by DNA sequencing.

### Western Blot Analysis and Immunofluorescent Staining

Human melanoma A2058 cells transfected with mSec 61-mCherry were fixed in 4% paraformaldehyde in PBS for 1 hour at room temperature, permeabilized in 0.2% Triton X-100 buffer for 20 minutes, and then blocked in PBS containing 0.2% goat serum. For immunofluorescence staining, cells were stained with primary antibody 1:200 at room temperature overnight. Slices were washed twice and incubated with secondary antibody before mounting with Antifade.

Equal amounts of proteins were loaded and separated on SDS-PAGE (4–12% SDS Bis Tris gels, ThermoFisher Scientific). Protein bands were transferred from the SDS-PAGE to a nitrocellulose membrane which were probed with primary and then secondary antibodies. Secondary antibodies were incubated for 1 h at room temperature. Chemiluminescence was visualized and exposed to film after washing.

### Imaging with VA-TIRFM and high-NA TIRF-SIM

To monitor MCSs dynamics, cells transfected with SNAPf-E-syt1/mCherry-Sec 61β/STIM1-EGFP or with G-CEPIA1er were initially bathed in Ca^2+^-free Hank’s Balanced Salt Solution (Ca^2+^-free HBSS, in mg/L: D-Glucose 1000, NaCl 8000, Na_2_HPO_4_ 48, NaHCO_3_ 350, KCl 400, and KH_2_PO_4_ 60, pH 7.4 adjusted with 1 mM NaOH). Under resting conditions, 150 frames of the images were taken at 5.28 Hz using VA-TIRFM or high-NA TIRF-SIM. Then, these cells were treated with 100 μm tBuBHQ for 5 min, and 150 frames at 5.28 Hz were obtained. After washing the tBuBHQ, we began to perform 200-frame imaging (5.28 Hz), and during the imaging, the incubation solution was changed to Ca^2+^-containing HBSS (in mg/L: D-Glucose 1000, CaCl_2_ 140, NaCl 8000, Na_2_HPO_4_ 48, NaHCO_3_ 350, KCl 400, KH_2_PO_4_ 60, MgCl_2_·6H_2_O 100, and MgSO_4_·7H_2_O 100, pH = 7.4 adjusted with 1 mM NaOH) by perfusion. We selected representative frames under each condition for presentation in the figures.

### Cytosolic and ER calcium imaging using wide-field microscopy

To image the cytosolic Ca^2+^, the cells were loaded with Cal-520-AM (5 μM) for 20 min at 37 °C. After three washes, we incubated the cells for another 20 min in fresh DMEM without phenol red at 37 °C. To image the whole-cell ER Ca^2+^, the cells were transfected with pCMV G-CEPIA1er. Then, these two cell types were transferred to Ca^2+^-free HBSS for time-lapse imaging at 2 Hz using wide-field microscopy. The entire imaging process included 1 min under resting conditions, 5 min of incubation with tBuBHQ, ~3 min of recovery after washing the tBuBHQ and another 5 min after changing to Ca^2+^-containing HBSS.

### VA-TIRFM and image reconstruction

The VA-TIRFM instrument was built on a commercial inverted Olympus IX81 microscope equipped with a TIRF objective (NA 1.7 Apo N100 × HO oil immersion lens, Olympus). Combined illumination solid state lasers (emission at 488 nm and 561 nm, Sapphire, coherent) controlled by an Acousto-Optical Tunable Filter (AOTF, AOTFnC 400–650, AA OPTO-ELECTRONIC) were projected onto the active area of a digital micro-mirror device (DMD, 0.7XGA, Texas Instruments) to generate different orientations and angles. The emitted fluorescence was captured by an EMCCD camera (iXon 897U, Andor). An OptoSplit (OptoSplit II, Cairn Optics) placed between the microscope and camera allowed us to image two spectral channels side-by-side on a single CCD chip. The entire system was controlled by Andor iQ commercial software. Typically, 10 raw images with incident angles of 48.4, 48.5, 48.6, 48.7, 48.9, 49.3, 49.8, 50.9, 52.8 and 60.9 degree were recorded at a frequency of 0.8 s/vol, which corresponded to TIRF penetration depths of 832.5, 488.1, 379.3, 321.0, 256.4, 195.4, 158.3, 119.6, 90.8 and 55.3 nm beneath the cell surface, respectively^15^.

With rapidly changing incident angles, the ensemble fluorescence intensities of molecules within the ER-PM contacts along axial-axis were measured multiple times. Having known different exponential dependences of fluorescence excitation at different excitation incident angles, we could solve the problem of the amount and the spatial distribution of fluorescent molecules within the volume of TIRF illumination. For the reconstruction of VA-TIRF data, we have used a well-established algorithm^16^. The accurate separation of fluorescence objects 40~50 nm axially apart has been validated with tilted coverslips coated with fluorescent beads under different incident angles of TIRF illumination, and also by the three-dimensional actin architecture and dynamics movements of TfnR-phluorin/Rab11A-mCherry labeled vesicles. Using the same technology, we have also visualized invagination of clathrin-coated pits in axial direction in our previous paper^15^, which also validated the method in dealing with biological samples.

### High-NA TIRF-SIM

We have recently published the detail of our high-NA TIRF-SIM elsewhere^14^. It was built on a commercial inverted fluorescence microscope (IX73, Olympus) equipped with a TIRF objective (Apo N 100X/1.7 HI Oil, Olympus), a multiband bandpass filter set (dichroic mirror: Di01-R405/488/561/635-25 × 36, Semrock; emission filter: FF01-446/523/600/677-25, Semrock) and a continuous autofocus system to maintain focus over time. We used two lasers (Sapphire 488LP-200, Coherent; and MRL-III -640-150 561LP-200, IL photonics) as light sources and an acoustic optical tunable filter (AOTF, AA Opto-Electronic, France) to combine, switch, and adjust the illumination power of the lasers. We used a ferroelectric liquid crystal on a silicon spatial light modulator (SLM, SXGA-3DM, Fourth Dimension Display) to generate the structure illumination patterns, which were formed by the interference of the ±1 order of the diffraction beam from the SLM. Then, the light passed through another lens (L3, AC254-125, Thorlabs) and a tube lens (L4, ITL200, Thorlabs) to focus on the back focal plane of the objective lens, which was interfered by the image plane after passing the objective lens. Emitted fluorescence collected by the same objective passed through a dichroic mirror (DM), an emission filter and another tube lens. Finally, the emitted fluorescence was split by an image splitter (W-VIEW GEMINI, Hamamatsu, Japan) before being captured by a sCMOS camera (Flash 4.0 V2, Hamamatsu, Japan). The time-lapse images were acquired using Hamamatsu’s image acquisition and analysis software suite HCImage.

### Estimation of axial movement of E-syt1

For an incident laser with a wavelength λ_*i*_ at incident angle $\,{\rm{\theta }}$, the intensity detected in the camera I_*θ*_ decays exponentially with distance z away from the interface,

$${{\rm{I}}}_{\theta }={I}_{0}\cdot {e}^{-z/d}$$

*I*_0_ is the intensity at the interface, d is the penetration depth,

$${\rm{d}}={{\rm{\lambda }}}_{i}/4\pi \cdot {({n}_{1}^{2}sin{\theta }^{2}-{n}_{2}^{2})}^{-\frac{1}{2}}$$

*n*_1_, *n*_2_ are the refractive indices of glass and the media at the interface, respectively.

The relative increases in fluorescence intensity $\frac{{I}_{\theta ,1}}{{I}_{\theta ,0}}$ at incident angle θ with distance *z*_1_, *z*_0_ away from interface is,

$$\frac{{I}_{\theta ,1}}{{I}_{\theta ,0}}=\frac{{I}_{0}\cdot {e}^{-{z}_{1}/d}}{{I}_{0}\cdot {e}^{-{z}_{0}/d}}={e}^{-({z}_{1}-{z}_{0})/d}$$

So,

$${\rm{\Delta }}z={z}_{1}-{z}_{0}=-dln\frac{{I}_{\theta ,1}}{{I}_{\theta ,0}}$$

Specifically, we imaged under the TIRF illumination at incident beam angle ${\rm{\theta }}\approx 59.65^\circ$ (penetration depth ${\rm{d}}=488/4\pi \cdot {({1.788}^{2}sin{\theta }^{2}-{1.333}^{2})}^{-\frac{1}{2}}\approx 50\,nm$). Therefore, a 27.35% increase of E-syt1 fluorescence corresponded to an axial movement of ${\rm{\Delta }}z=-50\times ln\frac{1+0.2735}{1}=-12.1\,{\rm{nm}}$.

### Image processing and data analysis

The particle size distribution, fluorescence intensity quantification and post-reconstruction image processing were primarily performed using Fiji software. The 3D segmentation and the rendering of ER-PM junctions were performed by the Amira (Thermo Fisher Scientific). Fluorescence images of ER marker were firstly imported into Amira, and then automatically segmented into labeled field by balanced histogram thresholding approaches with segmentation editor tool of Amira. Finally, we manually edited the features slice-by-slice and used the SurfaceGen module to generate the 3D rendered volume for data visualization and analysis.

Although super-resolution TIRF-SIM could resolve puncta or ring-shaped structures, we still used the corresponding conventional TIRF image stacks to determine their sub-pixel localizations. First, we removed the background from obtained raw image stack with the à trous wavelet transform algorithm^28^. Then we manually applied a threshold a bit lower to generate a mask to ensure all junction structures were included. After manually frame-by-frame confirmation to discard incorrect regions of interest, we used the weighted centroid method to calculate their localizations down to sub-pixel resolution. Both ring-shape and puncta-shape ER structures behave similarly under the TIRF microscope. The different localizations of individual ER structure at different time points were jointly connected using a multiple spots tracking method as reported previously^29^, which produced the trajectory map of all ER structures in time.

The schematic diagram was prepared using Illustrator (Adobe). The reconstruction of the images acquired by TIRF-SIM was processed using customized MATLAB software. The data were plotted using IGOR Pro software (WaveMetrics).

## Supplementary information

Video S1

Video S2

Video S3

Video S4

Video S5

Supplementary Information

## Data Availability

The datasets generated and/or analyzed during the current study are available from the corresponding author on reasonable request.
