# Supplementary material for: E-syt1 Re-arranges STIM1 Clusters to Stabilize Ring-shaped ER-PM Contact Sites and Accelerate Ca2+ Store Replenishment
Source: Sci Rep. 2019 Mar 8;9:3975. doi: 10.1038/s41598-019-40331-0 (PMC6408583; doi:10.1038/s41598-019-40331-0)
Supplement: Supplementary file 6 — Supplementary Information [file 41598_2019_40331_MOESM6_ESM.pdf]

Supplementary Information

E-syt1 Re-arranges STIM1 Clusters to Stabilize Ring-shaped ER-PM Contact Sites and Accelerate Ca^{2+} Store Replenishment

Fei Kang[1], Mengxuan Zhou[1], Xiaoshuai Huang[1], Junchao Fan[2], Lisi Wei[1], Jerome Boulanger[3], Zengzhen Liu[3], Jean Salamero[3], Yanmei Liu[1,*], Liangyi Chen[1,*]

[1]State Key Laboratory of Membrane Biology, Beijing Key Laboratory of Cardiometabolic Molecular Medicine, Institute of Molecular Medicine, Peking University, Beijing 100871, China

[2]Key Laboratory of Image Processing and Intelligent Control of Ministry of Education of China, School of Automation, Huazhong University of Science and Technology, Wuhan, 430074, China

[3]Institut Curie, PSL Research University, CNRS UMR 144 & Cell and Tissue Imaging Facility, Paris, France.

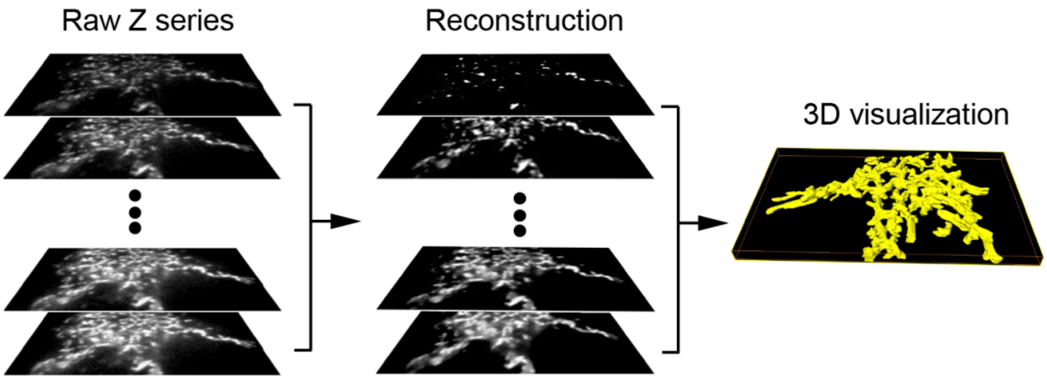

Figure S1 The workflow of the 3D reconstruction of multiple images of Sec61β in the HEK293 cell co-transfected with mCherry-Sec61β and STIM1-EGFP and imaged with VA-TIRFM.

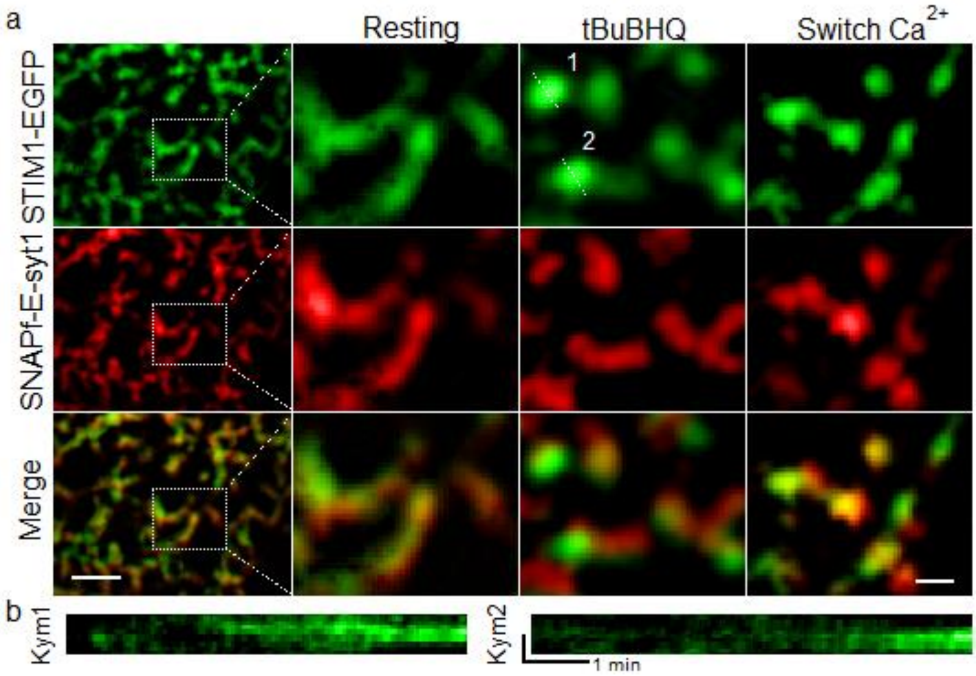

Figure S2 (**a**) STIM1 and E-syt1 mediated formation of punctated ER-PM contacts under conventional TIRF illumination. Representative TIRF images of cortical ER in a HEK293 cell co-transfected with STIM1-EGFP and SNAPf-E-syt1 under resting conditions, store depletion and store replenishment. This figure is representative of three independent experiments. Scale bars, left 2 μm; right 0.5 μm. (**b**) Kymographs of STIM1 aggregation induced by the ER store depletion. Vertical, scale bars, 0.5 μm.

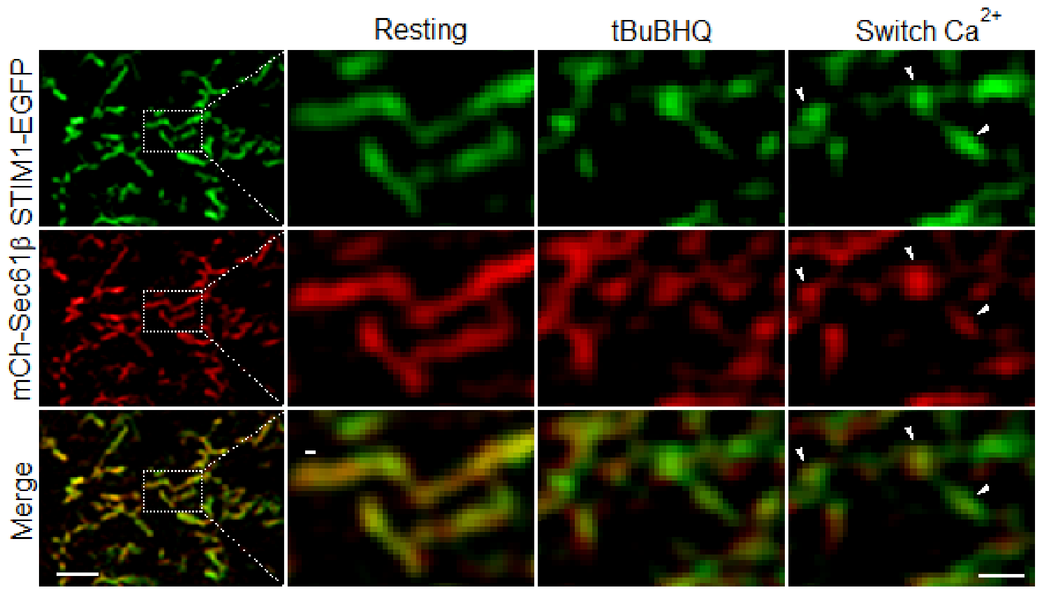

Figure S3 STIM1 alone induces formation of punctate ER-PM MCSs. Representative TIRF-SIM images of cortical ER in HEK293 cells co-transfected with STIM1-EGFP and mCherry-Sec61β under resting conditions, store depletion and during replenishment. This figure is representative of three independent experiments. Scale bars, left 2 μm; right 0.5 μm.

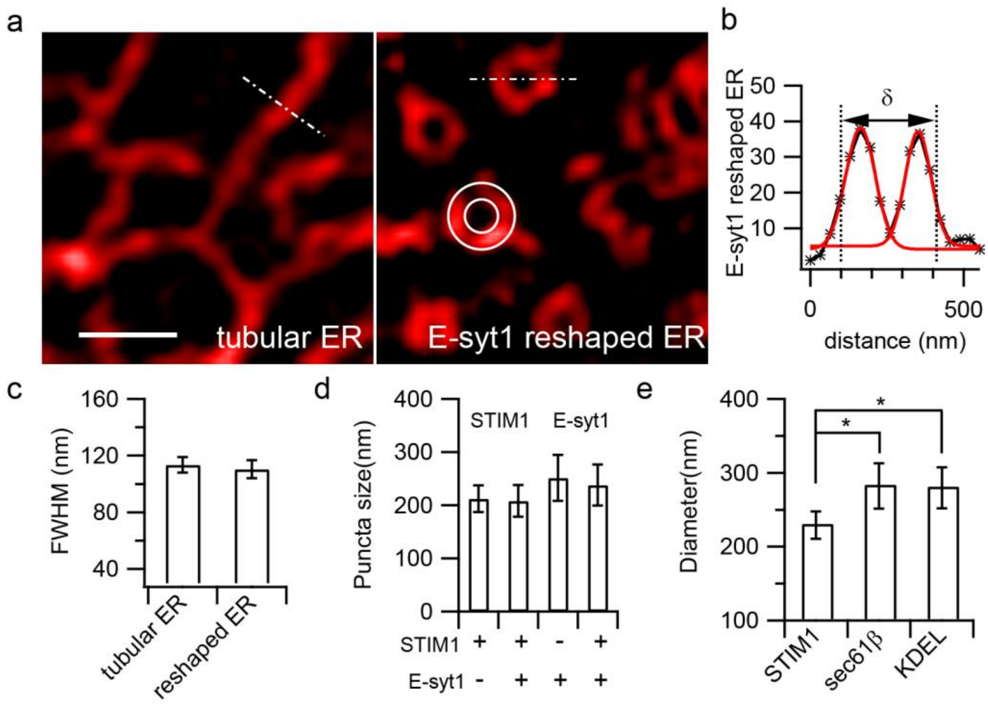

Figure S4 Quantification of the size of STIM1- and E-syt1-mediated MCSs under different conditions. (**a**) Representative TIRF-SIM images of ER tubules and ring-shaped MCSs in HEK293 cells co-transfected with mCherry-Sec61β and EGFP-E-syt1 under resting conditions (left), and during store replenishment (right). Scale bar, 2 μm. (**b**) Diameter of ring-shaped MCSs were determined by the width δ as shown in this figure. (**c**) Width measurements of tubular ER (n = 72)

and E-syt1-reshaped ER (n = 75). (**d**) Average sizes of STIM1 puncta (during store depletion, n = 67 and 75, respectively) and E-syt1 puncta (during store replenishment, n = 67 and 75, respectively) in HEK293 cells transfected with STIM1-EGFP or SNAPf-E-syt1 alone or both. (**e**) Average diameters of MCS rings labeled with different ER proteins (STIM1, n = 65; sec61β, n = 72, KDEL, n = 65). Five independent experiments, error bars show SEM.

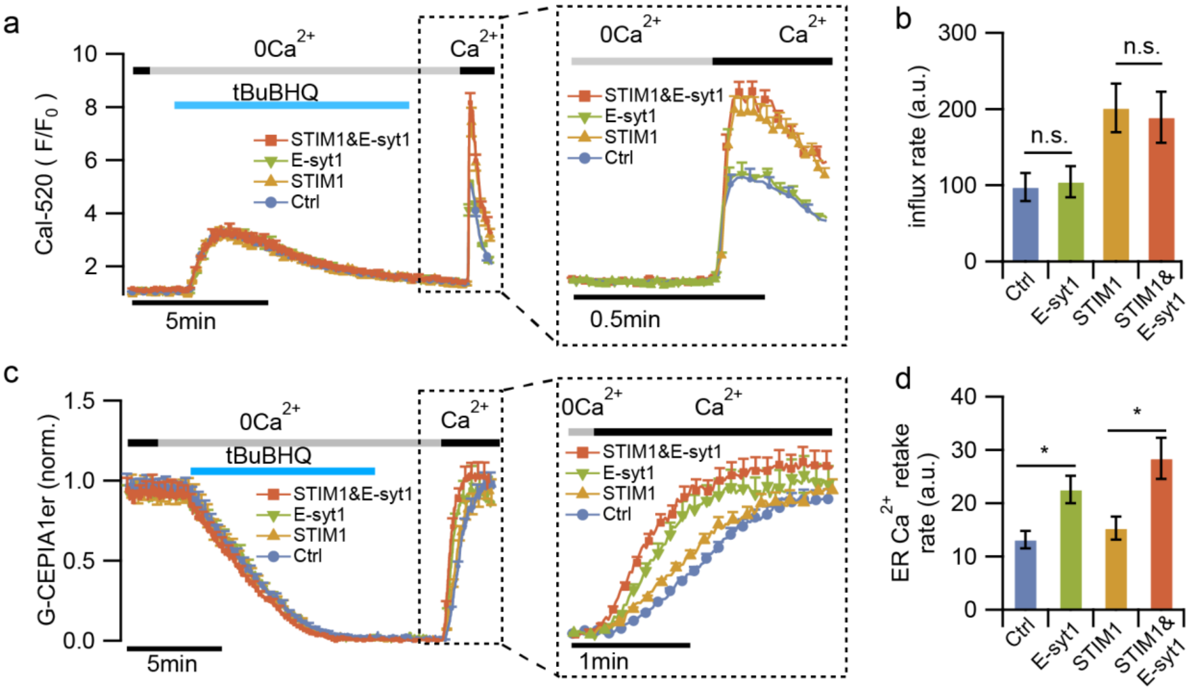

Figure S5 Overexpressing E-syt1 accelerates ER Ca^{2+} store recovery without affecting the Ca^{2+} influx via SOCE. (**a**) Time courses of the normalized fluorescence signals of Cal-520 in control cells and cells transfected with STIM1-EGFP or SNAPf-E-syt1 alone or both. We used tBuBHQ in 0 Ca^{2+} bath solution to deplete the ER Ca^{2+} store and switched the bath solution to Ca^{2+}-containing HBSS to initiate Ca^{2+} store replenishment. The boxed area in the left graph is enlarged in the right. (**b**) Maximum Ca^{2+} influx rates in cells in a calculated as the maximum slopes of the increase in Cal-520 fluorescence during store replenishment. (**c**) Time courses of the ER Ca^{2+} levels in control cells (transfected with G-CEPIA1er) and cells co-transfected with G-CEPIA1er and STIM1-mCherry or SNAPf-E-syt1 alone or both. Prior to the switching to Ca^{2+}-containing solution, we continuously perfused cells with Ca^{2+}-free solution for ~3 more minutes to remove tBuBHQ from the cellular environment and restore SERCA activity. (**d**) Maximum ER Ca^{2+} uptake rates in cells in (**c**). Mean ± SEM is shown (more than 35 cells for each, at least three independent experiments).

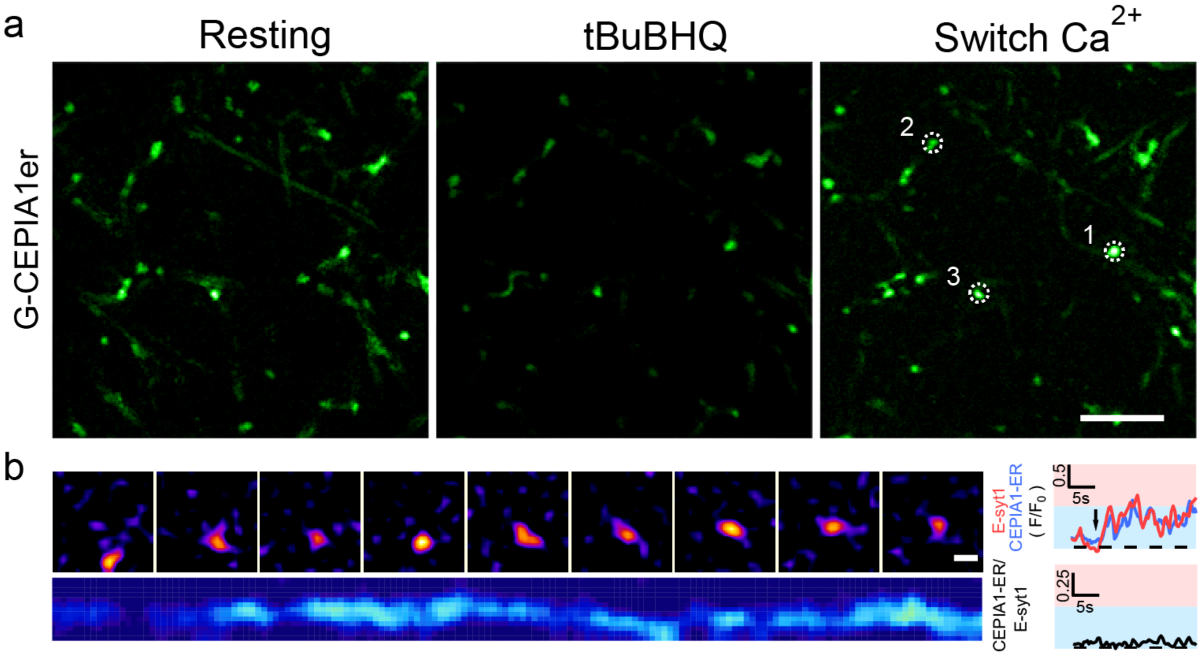

Figure S6 Mobile ER-PM MCSs experience little ER Ca^{2+} uptake during movement. (**a**) Representative TIRF-SIM images of cortical ER in a HEK293 cell co-transfected with SNAPf-E-syt1 and G-CEPIA1er under resting conditions, store depletion and during replenishment. (**b**) Representative example of the montage (top) and kymograph (bottom) of a mobile E-syt1 punctum. Time courses of the normalized fluorescence signals of G-CEPIA1er (blue) and E-syt1 (red) in the event shown on the right. The corresponding trace of G-CEPIA1er in reference to SNAPf-E-syt1 is shown in black. This data is representative of four independent experiments. Scale bars, **a**) 2 μm; **b**) 0.25 μm.

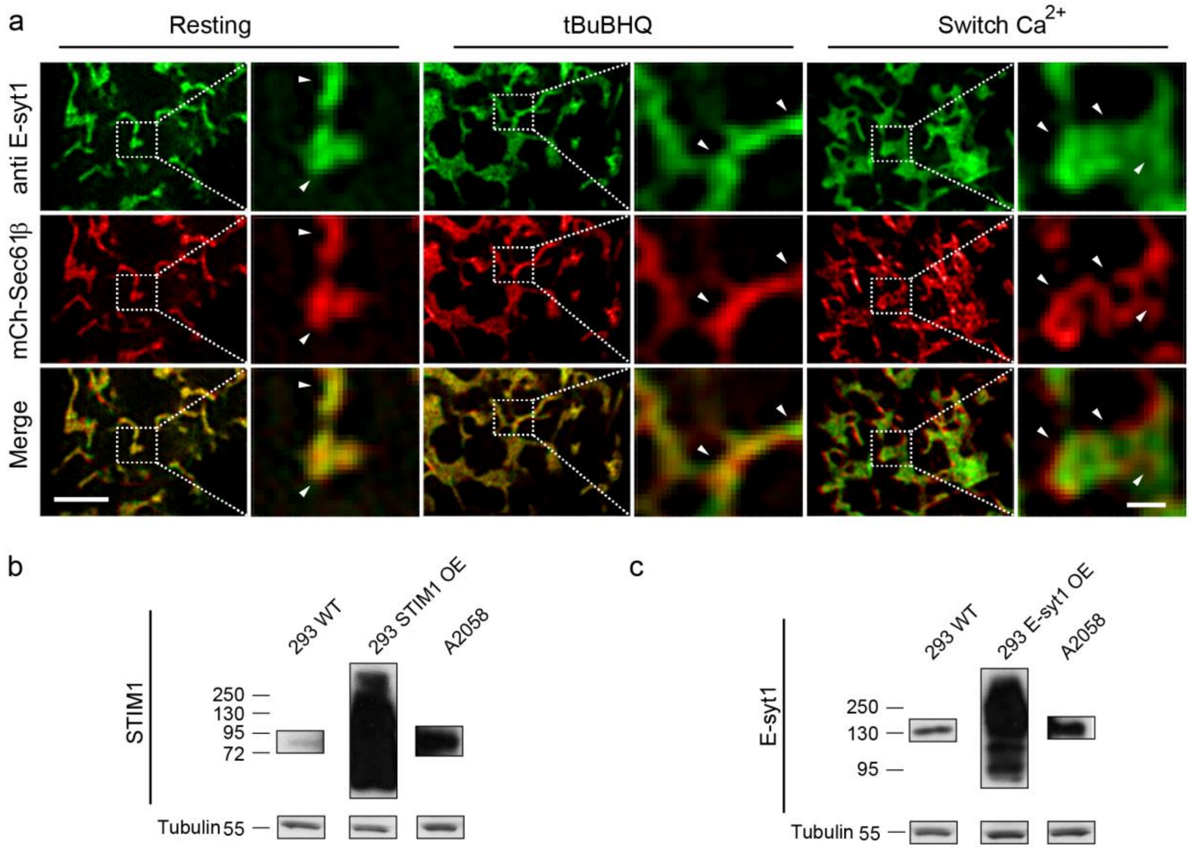

Figure S7 Ring-shaped structures physiologically exist in human melanoma A2058 cells in which E-syt1 is highly expressed. (**a**) Immunofluorescent staining assay of A2058 cells shows ring-shaped structures during SOCE. This data is representative of three independent experiments.Scale bars, left 2 μm; right 0.5 μm. (**b**) (**c**) Western-blot assay detected the abundance of STIM1 and E-syt1 in HEK293 cells, HEK293 cells transfected with STIM1-EGFP/EGFP-E-syt1 and A2058 cells. Tubulin was used as a loading control. Full-length blots are presented in Figure S8.

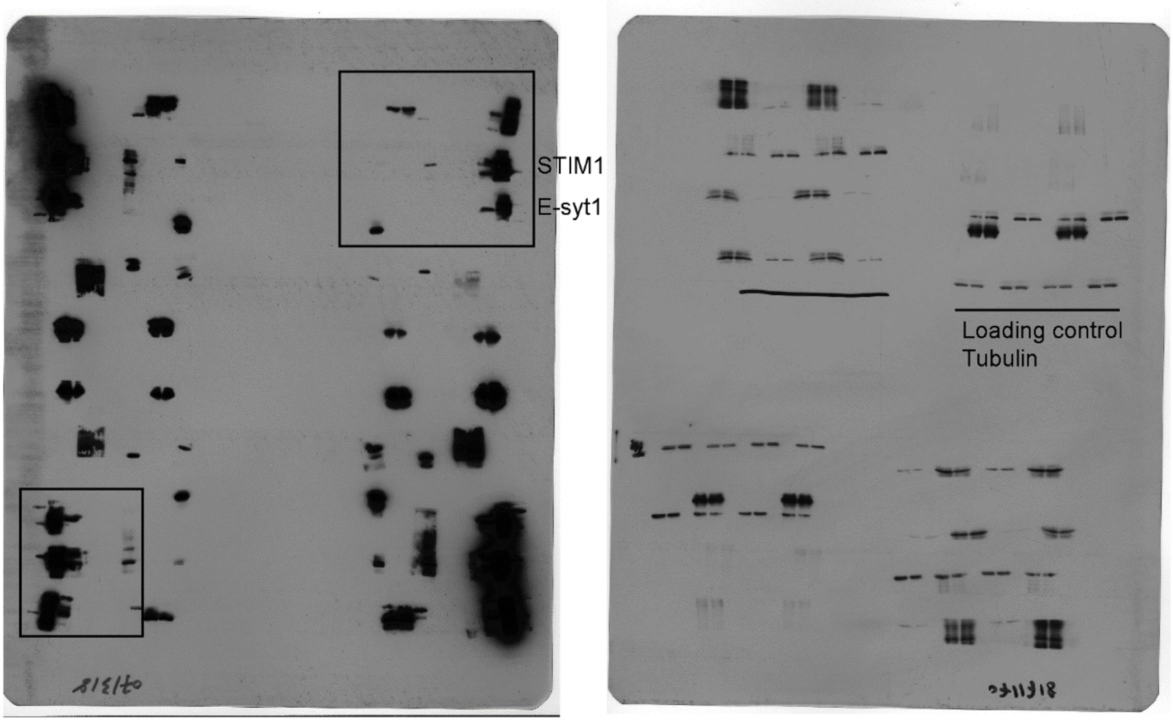

Figure S8 Full picture of Western blotting for detecting the abundance of STIM1 and E-syt1 in HEK293 cells, HEK293 cells transfected with STIM1-EGFP/EGFP-E-syt1 and A2058 cells.

Supplementary Video S1 3D volumetric rendering of STIM1-EGFP labeled ER structures in HEK293 cell co-transfected with mCherry-Sec61β and STIM1-EGFP after being treated with tBuBHQ.

Supplementary Video S2 Formation of ring-shaped ER-PM STIM1-MCSs triggered by the activation of SOCE and formation of E-syt1 puncta. The HEK293 cell was co-transfected with SNAPf-E-syt1 and STIM1-EGFP, and observed under the TIRF-SIM. Left: STIM1-EGFP (fire); middle: SNAPf-E-syt1 (red); right: the top plots the change of fluorescent intensity of the E-syt1 punctum indicated by arrow. The kymograph of STIM1-EGFP is shown at the bottom, which shows the formation of ring-shaped ER-PM contacts. Scale bar, 0.5 μm.

Supplementary Video S3 Formation of ring-shaped ER-PM Sec61β-MCSs triggered by the activation of SOCE and formation of E-syt1 puncta. The HEK293 cell was co-transfected with EGFP-E-syt1 and mCherry-Sec61β, and observed under the TIRF-SIM. Left: mCherry-Sec61β (fire); middle: EGFP-E-syt1 (red); right: the top plots the change of fluorescent intensity of the E-syt1 punctum indicated by arrow. The kymograph of mCherry-Sec61β is shown at the bottom, which shows the formation of ring-shaped ER-PM contacts. Scale bar, 0.5 μm.

Supplementary Video S4 Formation of ring-shaped ER-PM KDEL-MCSs triggered by the activation of SOCE and formation of E-syt1 puncta. The HEK293 cell was co-transfected with SNAPf-E-syt1 and EGFP-KDEL, and observed under the TIRF-SIM. Left: EGFP-KDEL (fire); middle: EGFP-E-syt1 (red); right: the top plots the change of fluorescent intensity of the E-syt1 punctum indicated by arrow. The kymograph of EGFP-KDEL is shown at the bottom, which shows the formation of ring-shaped ER-PM contacts. Scale bar, 0.5 μm.

Supplementary Video S5 Trajectories of an ER-PM MCS during the ER store depletion and replenishment via SOCE. The HEK293 cell was co-transfected with SNAPf-E-syt1 and STIM1-EGFP, and observed under the TIRF-SIM. We applied tBuBHQ in Ca^{2+}-free solution at 1 min to deplete the ER Ca^{2+} store, and switched the solution to a Ca^{2+}-containing bath solution to enable extracellular Ca^{2+} entry via SOCE. Left: STIM1-EGFP (fire); right: SNAPf-E-syt1 (red). Scale bar, 0.5 μm.
